# Clinical Diagnosis and Novel Treatment of Eyelid Molluscum Contagiosum

**DOI:** 10.1155/crdi/9730930

**Published:** 2025-04-28

**Authors:** Bangtao Yao, Yinling Wu, Chang'an Hu, Bei Wang, Xiaoli Yue

**Affiliations:** ^1^Department of Ophthalmology, Nanjing Lishui People's Hospital, Zhongda Hospital Lishui Branch, Southeast University, Nanjing, China; ^2^Department of Endocrinology, Nanjing Lishui People's Hospital, Zhongda Hospital Lishui Branch, Southeast University, Nanjing, China; ^3^Department of Otolaryngology, Head and Neck Surgery, Nanjing Lishui People's Hospital, Zhongda Hospital Lishui Branch, Southeast University, Nanjing, China; ^4^Department of Pathology, Nanjing Lishui People's Hospital, Zhongda Hospital Lishui Branch, Southeast University, Nanjing, China

**Keywords:** curettage, diagnosis, eyelid, molluscum contagiosum, treatment

## Abstract

Molluscum contagiosum is an infectious skin disease caused by the molluscum contagiosum virus that affects 6 million people in the United States annually. However, molluscum contagiosum on the eyelid is uncommon. The aim of this article is to describe the clinical diagnosis and effect of curettage using fine forceps under an operating microscope in a patient with eyelid molluscum contagiosum. We reported a patient who presented with a two-month long history of a dome-shaped skin-colored papule with a central umbilication containing white caseous material on the left upper eyelid margin. The patient underwent a complete curettage using fine forceps under an operating microscope. Postoperative histopathology revealed Henderson–Patterson bodies. A diagnosis of eyelid molluscum contagiosum was made. At the 1-week follow-up, his symptoms had resolved completely without scarring. No recurrence was observed at a nine-month follow-up. This novel surgical method may be beneficial and safe for patients with eyelid molluscum contagiosum. Slit-lamp examination is a noninvasive and valuable tool for evaluating this condition. Typical pathological features may help in the diagnosis. Early diagnosis and precise treatment can prevent reinfection and control transmission. This report is particularly relevant to dermatologists and ophthalmologists because it offers valuable insights into a rare localization of molluscum contagiosum and its treatment approach.

## 1. Introduction

Molluscum contagiosum is an infectious skin disease caused by the molluscum contagiosum virus that affects 6 million people in the United States annually. Certain groups, such as sexually active adults, immunosuppressed patients, and children, are particularly prone to this infection [1]. Typically, autoinoculation occurs more frequently than transmission to other individuals. Molluscum contagiosum mainly occurs on the face, torso, extremities, and genitals. It is usually transmitted through sexual or physical contact or unsanitary environment [2–4].

Ocular molluscum contagiosum (e.g. eyelid, conjunctiva, and cornea) is uncommon [2, 4]. However, the exact prevalence of eyelid involvement has not been described. The diagnosis and treatment of molluscum contagiosum in delicate areas such as the eyelids can be challenging [4–6]. Typical pathological features may help in the diagnosis [5]. This novel report describes the clinical diagnosis and effect of curettage using fine forceps under an operating microscope in a patient diagnosed with eyelid molluscum contagiosum.

## 2. Case Presentation

**Figure 1:**
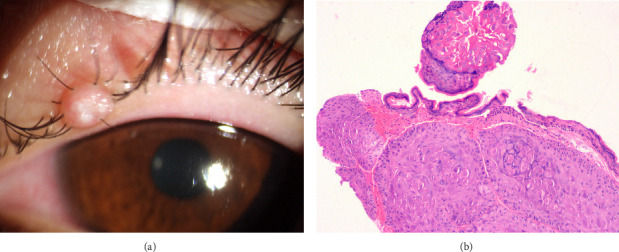
(a) Slit-lamp examination revealed a dome-shaped skin-colored papule (4 × 4 mm) with a central umbilication containing white caseous material on the left upper eyelid margin. (b) Postoperative histopathology revealed large eosinophilic cytoplasmic inclusion bodies (Henderson–Patterson bodies). Hematoxylin and eosin ×100 magnification.

A 25-year-old Chinese man presented with a 2-month history of persistent discomfort on the left eyelid. The patient denied any history of ocular or systemic diseases. He had received unsuccessful treatment with topical corticosteroids and antibiotic for 1 month. Slit-lamp examination revealed a dome-shaped skin-colored papule (4 × 4 mm) with a central umbilication containing white caseous material on the left upper eyelid margin (Figure 1(a)). Anterior segment optical coherence tomography revealed a hyperreflective elevation in the corresponding area. The remaining ocular examinations did not reveal any abnormalities. Inspection of the face, torso, extremities, and genital region was unremarkable. Therefore, the patient requested surgical treatment. Further examinations, including routine blood, human immunodeficiency virus, and *Treponema pallidum* tests, were negative. A provisional diagnosis of eyelid molluscum contagiosum was made. After injecting 1 cc of 2% lidocaine around the lesion as local anesthesia, the patient underwent a complete curettage using fine forceps under an operating microscope. Postoperative histopathology revealed large eosinophilic cytoplasmic inclusion bodies (Henderson–Patterson bodies) (Figure 1(b)).

Based on the above findings, eyelid molluscum contagiosum was finally diagnosed. Tobramycin eye ointment was applied to the lesion twice daily for 7 days. The patient was advised to avoid skin contact with family members; his towels and sheets were maintained separately.

**Figure 2:**
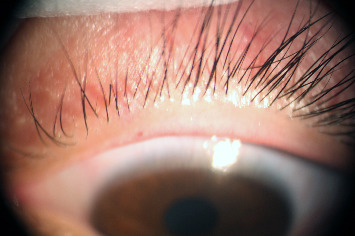
At the 1-week follow-up, his symptoms had resolved completely without scarring.

At the 1-week follow-up, his symptoms had resolved completely without scarring (Figure 2). No recurrence was observed at the one-month follow-up and a further nine-month follow-up.

## 3. Discussion

In clinical practice, misdiagnosis of molluscum contagiosum as conjunctivitis is commonly reported because patients may present with red eye, especially in initial visit [7]. Slit-lamp examination and dermatoscopy are noninvasive and valuable methods for evaluating molluscum contagiosum [4, 8]. Slit-lamp examination shows a smooth, dome-shaped, skin-colored papule with a central umbilication containing white caseous material [4]. Dermatoscopy reveals a characteristic pattern of yellowish or white polylobular structures with peripheral vessels [8]. These features can help differentiate other eyelid conditions, such as eczema, basal cell carcinoma, chalazion, papilloma, histoplasmosis, sebaceous cysts, keratoacanthoma, and cryptococcosis [4]. Clinically, a definitive diagnosis relies on clinical examination and postoperative histopathology.

In some healthy adults, molluscum contagiosum may be self-limiting without intervention [9]. However, some untreated cases develop follicular conjunctivitis, punctate keratitis, and corneal neovascularization. Molluscum contagiosum can even persist symptomatically for months to years [4, 8].

Topical drugs (e.g. imiquimod (5%), salicylic acid, and tretinoin) are used to treat eyelid molluscum contagiosum; however, complications such as scarring and pigmentary changes should be considered [4]. The application of cantharidin (0.7%) and berdazimer gel (10.3%) has been proven to be well-tolerated and effective; however, these treatments should be avoided for eyelid margin lesions because the proximity to the conjunctiva and eyeball is sensitive and may result in irritation of these structures [1, 9]. Topical ganciclovir is ineffective against eyelid lesions, and corticosteroids may exacerbate this condition [4, 5]. Surgical methods include excision, curettage, cryotherapy, laser, and intralesional immunotherapy; however, destructive methods may result in the Koebner phenomenon and autoinoculation due to viral shedding [10]. Curettage is one of the most commonly used and effective methods for treating molluscum contagiosum. However, performing traditional curettage (a locally destructive therapy without a microscope) in such a delicate area (eyelid margin) is challenging because of proximity to the eye [11]. The procedure of curettage with fine forceps under an operating microscope mainly includes the following: (1) expose the surgical field adequately and adjust the microscope to a proper magnification; (2) use the fine forceps to apply gentle traction for elevating the bottom of eyelid molluscum contagiosum; and (3) smoothly scrape the lesion from the edge toward the center using a curette until the central umbilication is completely removed. The operating microscope provides high-magnification visualization, enabling the surgeon to clearly identify the structures and boundaries of the eyelid lesions [12]. Compared with traditional curettage, our technique can precisely remove the eyelid lesions, reduce complications, and achieve faster recovery. Thus, it may be beneficial and safe for patients with molluscum contagiosum, particularly for eyelid or recurrent cases. In this case, we applied this technique and obtained favorable outcomes. Furthermore, patients should be encouraged to avoid scratching lesions or sharing towels and sheets with close contacts [4].

## 4. Conclusion

Herein, we describe the clinical diagnosis and the effect of curettage with fine forceps under an operating microscope in a patient with an unusual presentation of molluscum contagiosum. Early diagnosis and precise treatment can prevent reinfection and control transmission. This report is particularly relevant to dermatologists and ophthalmologists because it offers valuable insights into a rare localization of molluscum contagiosum and its treatment approach.

## Data Availability

The data that support the findings of this study are available on request from the corresponding author.
